# Potassium Vanadium Fluorides as Positive Electrode Materials for K‑ion Batteries

**DOI:** 10.1021/acsami.5c02298

**Published:** 2025-05-07

**Authors:** Kazushi Magara, Tomooki Hosaka, Ryoichi Tatara, Shinichi Komaba

**Affiliations:** Department of Applied Chemistry, 26413 Tokyo University of Science, 1-3 Kagurazaka, Shinjuku, Tokyo 162-8601, Japan

**Keywords:** rechargeable batteries, potassium-ion batteries, positive electrode materials, potassium vanadium fluoride, electrochemistry, potassium insertion materials

## Abstract

Potassium vanadium fluorides of K_5_V_3_F_14_, K_3_VF_6_, and KVF_4_ have been investigated as potential positive electrode materials for potassium-ion batteries. K_5_V_3_F_14_ and KVF_4_ exhibit low electrochemical activity with an ∼50 mAh g^–1^ initial capacity. By contrast, K_3_VF_6_ exhibits a promising electrochemical performance, displaying a voltage plateau at 3.7 V and a large initial capacity of 95 mA h g^–1^. A reversible V^4+/3+^ one-electron process is evidenced by X-ray absorption spectroscopy, whereas oxidation from V^4+^ to V^5+^ (from K_2_V^4+^F_6_ to K_1_V^5+^F_6_) is mainly irreversible and V^3+/2+^ (from K_3_V^3+^F_6_ to K_4_V^2+^F_6_) is almost inactive. K_3_VF_6_ displays partially irreversible structural changes during potassium extraction/insertion, especially when more than 1 mol of potassium ions are extracted from K_3_VF_6_. These results suggest that structural stability after potassium extraction is a key factor in the design of high-capacity fluoride materials.

## Graphical abstract

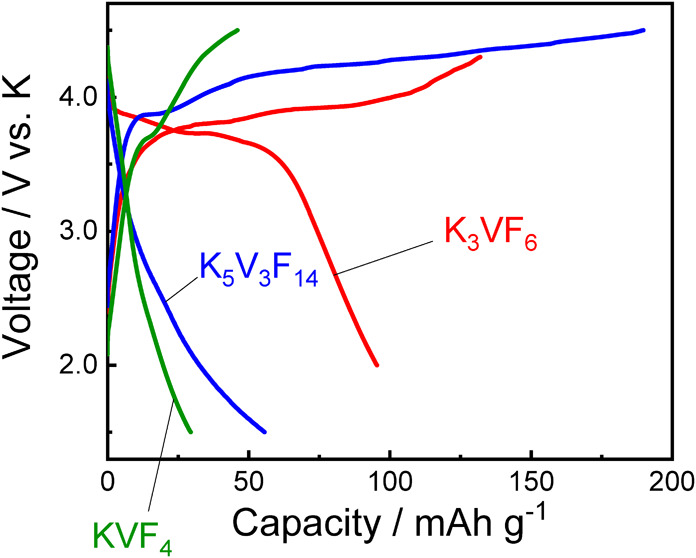

## Introduction

Lithium-ion batteries (LIBs) are predominantly used in portable devices, electric vehicles, and large-scale energy storage systems because of their high capacity, long cycle life, and high-power density.^1,2^ However, the scarcity (∼20 ppm in the Earth’s crust) and uneven distribution of lithium raw materials^3^ imply that the future demand may not be fulfilled. Potassium-ion chemistry is an alternative to lithium-ion because the potassium resource is abundant (>20000 ppm) in the Earth’s crust. Moreover, the lower standard electrode potential of potassium metal in organic electrolytes will assist in realizing high-voltage operation, comparable to that of LIBs.^4,5^ Thus, potassium-ion batteries (KIBs) have attracted considerable attention. A variety of materials have been used as positive electrode materials for KIBs, including layered transition metal oxides,^6^ Prussian blue analogs,^7,8^ and polyanionic compounds.^9–11^

Although layered oxides display high theoretical capacity, the actual capacity is usually limited by irreversible phase transitions and large voltage changes during potassium insertion/extraction, as observed in K_*x*_CoO_2_.^12,13^ Furthermore, most layered 3d transition metal oxides operate at low working potentials, which lowers the energy density.^14^ By contrast, Prussian blue analogues and polyanionic compounds have three-dimensional frameworks, which can mitigate irreversible phase transitions during potassium insertion and extraction. Moreover, some polyanionic compounds exhibit a high average working potential owing to the inductive effect of anionic species.^15^ For example, KTiOPO_4_-type KVOPO_4_,^10^ KVPO_4_F,^10,16,17^ and K_6_(VO)_2_(V_2_O_3_)_2_(PO_4_)_4_(P_2_O_7_)^18^ have been reported as 4 V-class positive electrode materials. However, the redox inactive polyanionic framework lowers the gravimetric and volumetric capacity, and the actual capacity nears the theoretical capacity.^11,19^

This study focused on fluoride-based materials, which usually have a three-dimensional (3D) framework that excludes heavy polyanionic ions, a scenario that would be advantageous to both the structural stability and specific capacity. Moreover, fluoride-based materials are expected to operate at higher potentials than metal-layered oxides owing to the inductive effect. The only fluoride-based positive electrode materials reported for KIBs are perovskite-type KMeF_3_ (Me = Fe, Mn) compounds,^20,21^ and materials in which some of the Mn (5%) is replaced with Co or Ni.^22,23^ However, their average working potentials are lower than 3 V; the low working potentials would be due to the low redox potential of the Fe^2+/3+^ couple and the low redox activity of other transition metals.^20–23^ Here, we focused on potassium-containing vanadium fluorides (K_*x*_V_*y*_F_*z*_) because vanadium typically displays a high redox potential with multivalent redox, which may lead to a high-potential operation and high capacity.

## Experimental Section

### Synthesis

K_3_VF_6_ ^24^ and K_3_V_0.75_Al_0.25_F_6_ were synthesized using a mechanochemical method. For the synthesis of carbon-coated K_3_VF_6_ (K_3_VF_6_@C), 0.3348 g (bulk volume of ∼0.29 mL) KF (Wako Pure Chemical Industries), and 0.2065 g VF_3_ (∼0.31 mL) (Kojundo Chemical Laboratory) were ball-milled with 10 wt % KB (∼1.5 mL) using a planetary ball mill (FRITSCH, P-7) with a 45 mL ZrO_2_ pot and balls at 600 rpm for 12 h (1 h milling and 15 min rest × 12 times). We used five φ5 mm and ∼250 φ2 mm balls, whose mass and bulk volume were 14.0 g and 4.1 mL, respectively. For the synthesis of carbon-coated K_3_V_0.75_Al_0.25_F_6_ (K_3_V_0.75_Al_0.25_F_6_@C,) 0.3407 g KF, 0.1583 g VF_3_, and 0.04104 g (∼0.97 mL) AlF_3_ (Kojundo Chemical Laboratory) were ball-milled with 10 wt % KB. After the synthesis, the samples were calcined in an Ar atmosphere (flow rate of 5 mL min^–1^) at 300 °C for 12 h to enhance their crystallinity.

K_5_V_3_F_14_ ^25^ and KVF_4_ ^25^ were synthesized *via* solid-state reactions. In detail, carbon-coated K_5_V_3_F_14_ (K_5_V_3_F_14_@C) was synthesized by mixing 0.2553 g KF (Wako Pure Chemical Industries, Ltd.) and 0.2846 g VF_3_ (Kojundo Chemical Laboratory) with 15 wt % KB. The mixture was calcined at 650 °C for 12 h under an Ar atmosphere (flow rate of 5 mL min^–1^). Carbon-coated KVF_4_ (KVF_4_@C) was synthesized from a powdery mixture of 0.1890 g KF (Wako Pure Chemical Industries, Ltd.) and 0.3510 g VF_3_ (Kojundo Chemical Laboratory) with 20 wt % KB. The mixture was calcined at 550 °C for 12 h under an Ar atmosphere.

### Electrochemical Measurement

The positive electrodes comprised 80 wt % active material, 10 wt % KB, and 10 wt % poly­(vinylidene fluoride) (PVdF). An optimum amount of N-methylpyrrolidone (Kanto Chemical) was used to prepare a uniform slurry. The slurry was coated on Al foil and dried at 100 °C under a vacuum. The counter electrode was metallic potassium (Aldrich). The electrolyte solution was 1.0 mol dm^–3^ KPF_6_ dissolved in EC/PC (1:1 v/v) solvent (Kishida Chemical) or 5.6 mol kg^–1^ KN­(SO_2_F)_2_ (Solvionic) (KFSA)/triglyme (G3) electrolyte (Kishida Chemical).

The Li and Na half-cells were fabricated by using 1.0 mol dm^–3^ LiPF_6_ in EC/DMC (1:1 v/v) and 1.0 mol dm^–3^ NaPF_6_ in EC/PC (1:1 v/v) electrolytes, respectively. A glass fiber filter (GB100R, ADVANTEC) was used as the separator. R2032-type coin cells were assembled in an Ar-filled glovebox (dew point: < −90 °C). The coin cells were cycled in the voltage range between 2.0 and 4.3 V at a rate of C/20, C/10, C/5, C/2, and 1 C and 2.0 and 4.6 V at a rate of C/10 for K_3_VF_6_ (1 C = 190 mA g^–1^). For K_5_V_3_F_14_, the coin cells were cycled in the voltage range between 1.5–4.5 V at a rate of C/10 (1 C = 218.1 mA g^–1^); the same voltage range and rate as those used for K_5_V_3_F_14_ were used for KVF_4_ (1 C = 161.4 mA g^–1^). All electrochemical tests are conducted at 25 °C. The current density and specific density were calculated based on the mass of active material, including carbon coating.

### Materials Characterization

The crystal structures of the obtained samples were elucidated by using X-ray diffraction (XRD) with Cu Kα radiation (Rigaku, SmartLab). For the XRD measurements, an in-house airtight sample holder was used to avoid exposing the sample to air. Synchrotron X-ray diffraction measurements were performed at the BL02B2 beamline^26^ in SPring-8, synchrotron facility, Japan at a wavelength of λ = 0.8 Å. The samples were ground and filled in a glass capillary tube (diameter = 0.3 or 0.5 mm) and sealed with resin in an Ar-filled glovebox to prevent exposing the electrodes to moisture. Rietveld and Le Bail analyses were performed using the RIETAN-FP^27^ and FOX programs,^28^ respectively. Schematic illustrations of the crystal structures were prepared using the VESTA program.^29^ The bond valence sum (BVS) of the elements was also calculated using the Bond_Str program inside FullProf Suite.^30^ Scanning electron microscopy (SEM; JCM-6000, JEOL Ltd.) was used to characterize the morphology of the synthesized powder. The elements on the surface of the K_3_VF_6_ electrode were identified by using energy-dispersive X-ray spectroscopy (EDS; JED-2300, JEOL Ltd.).

X-ray absorption spectroscopy (XAS) was performed at the BL14B2 beamline in SPring-8, Japan. For the XAS measurements, the cycled electrodes were sealed in a water-resistant polymer film inside an Ar-filled glovebox to minimize exposing the electrodes to moisture. XAS spectra were collected by using a silicon monochromator in transmission mode. The intensities of the incident and transmitted X-rays were measured using an ionization chamber at 298 K. The XAS spectra were analyzed using the ATHENA software package based on IFEFFIT.^31^

## Results and Discussion

### Structure and Morphology of K_5_V_3_F_14_, K_3_VF_6_, and KVF_4_

To choose promising candidates, we first created a ternary phase diagram of the K–V–F systems considering their theoretical capacity based on the potassium content and oxidation state of V (Figure 1a,b). Generally, in order to assemble K-ion batteries by combining K–V–F and graphite as the positive and negative electrodes, respectively, potassium-containing (fully discharged) materials are necessary for the positive electrode. Furthermore, based on our experience and knowledge of solid-state redox of V­(3/4/5+) in Li and Na-insertion materials,^15^ we reasonably focus on the tie line of V^3+^ in the phase diagram, i.e., the series of potassium vanadium fluorides of K_*y*_V­(III)_*z*_F_3z+y_. Namely, trivalent compounds K_5_V_3_F_14_, K_3_VF_6_, and KVF_4_ were selected because of the good balance of the theoretical capacity and feasibility of synthesis.

**Figure 1:**
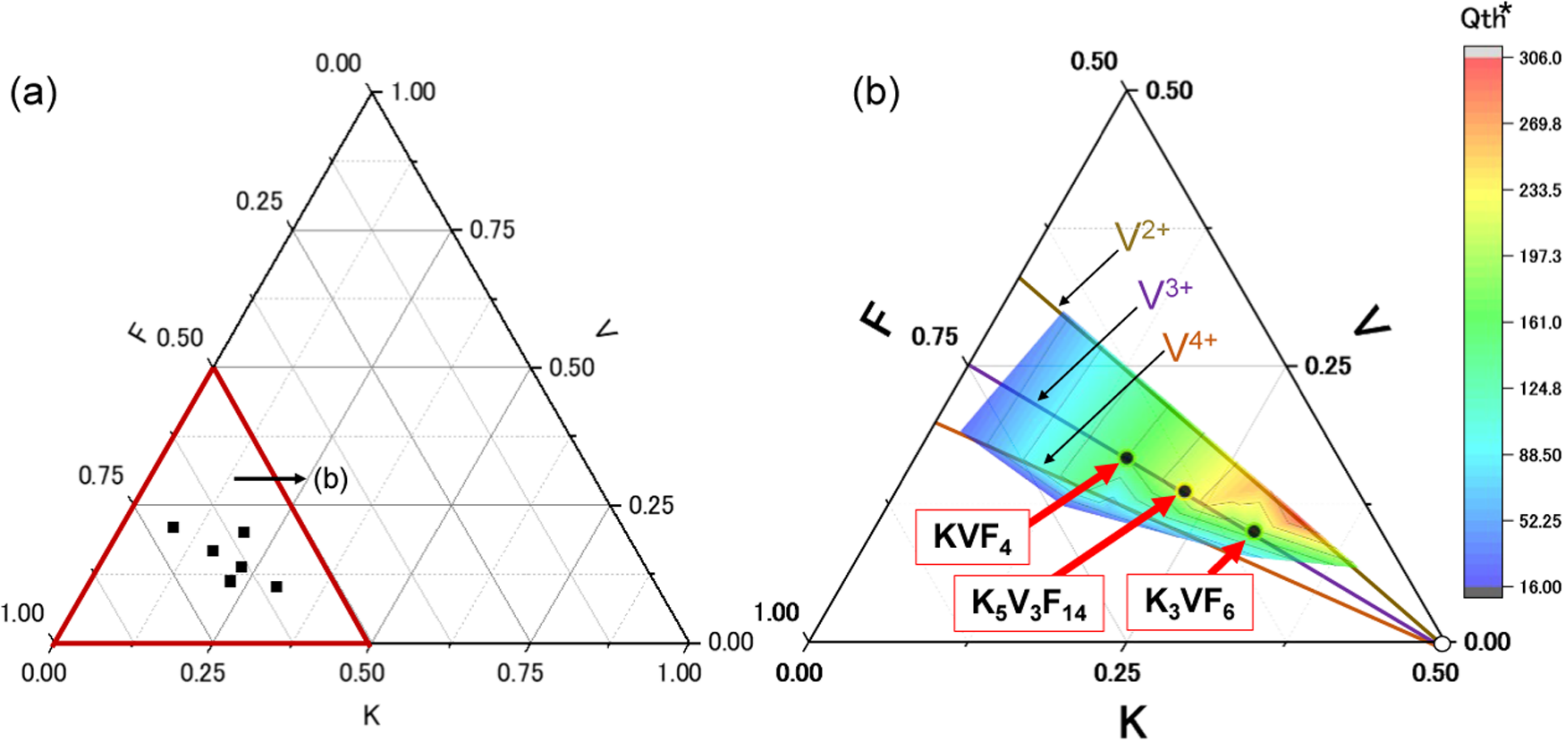
(a) Triangle phase diagram of K–V–F systems and (b) enlarged figure in the range of K = 0–0.5, V = 0–0.5, and F = 0.5–1 considering their calculated theoretical capacities, *Q*_th_, which are a smaller value of either theoretical capacity based on potassium content, i.e., full potassium extraction, or vanadium valence by assuming electrochemical oxidation of vanadium up to +5 from the initial state.

The SXRD pattern of synthesized K_3_VF_6_ is shown in Figure 2a, and the refined structural parameters are listed in Table S1. The main diffraction peaks could be indexed to the K_3_VF_6_ phase with a tetragonal space group of *I*4̅ /*m* (87), which is isostructural to β-K_3_AlF_6_.^32^ While a previous study reported *I*4̅ _1_ */a* (88) as a space group for a tetragonal-K_3_VF_6_, a better fitting was obtained with *I*4̅ /*m* space group for our sample (Figure S1).^33^ In addition to the main K_3_VF_6_ phase, the VO phase, which is contained in the raw material of VF_3_ as an impurity of ∼5% (Figure S2), was detected. The *R*_wp_ value was 6.18%, indicating a reasonable fitting. Moreover, the K/V ratio was estimated to be 3.08 by ICP-AES. These results showed that tetragonal-K_3_VF_6_ was successfully synthesized. Further reduction of the impurity is not only essential but also challenging due to the difficulty of removing the vanadium oxide(s). Because NaCl-type VO is electrochemically inactive due to its negligible potassium-ion conductivity, we used synthesized K_3_VF_6_ without further purification.

**Figure 2:**
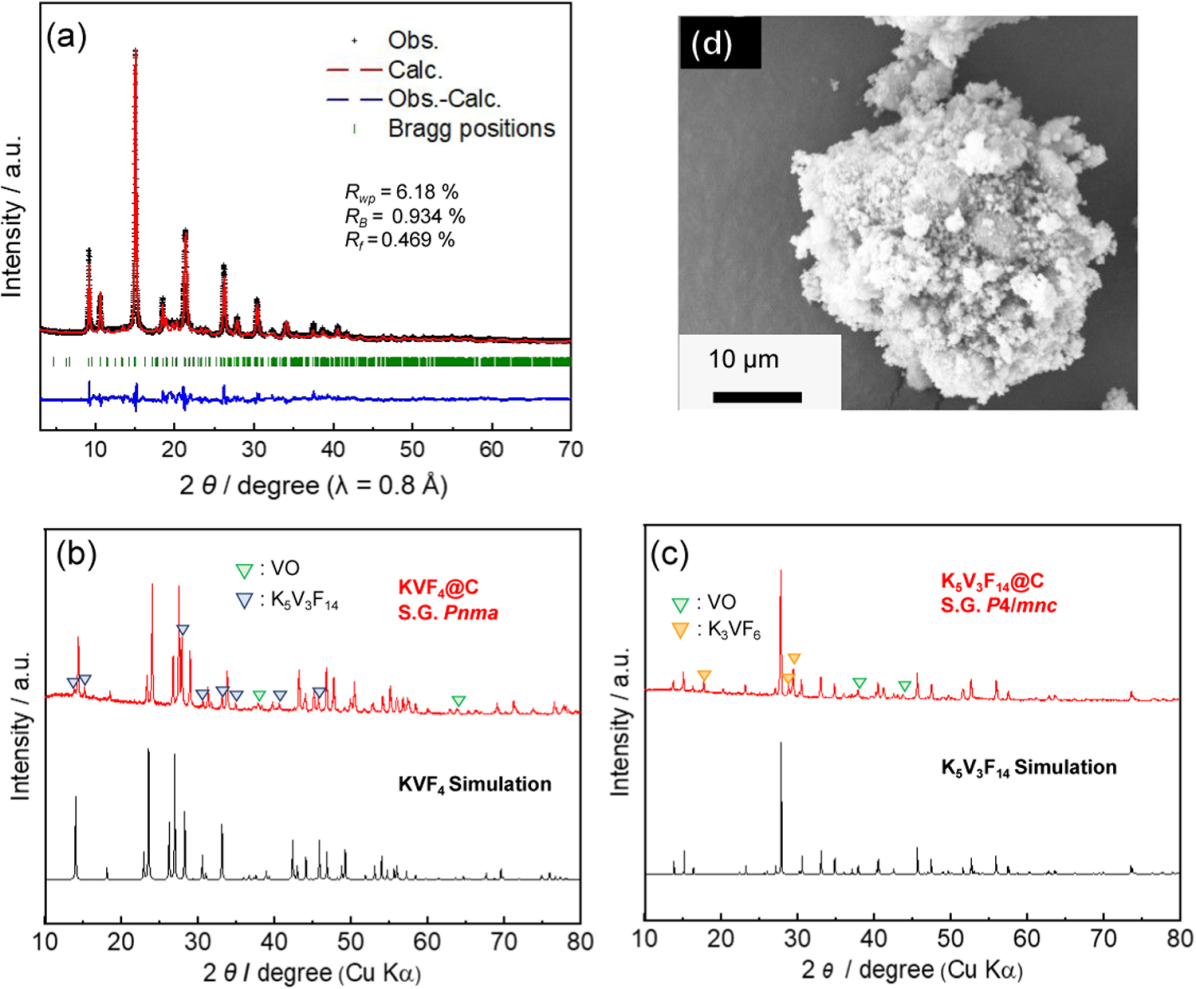
(a) Synchrotron X-ray diffraction pattern of the K_3_VF_6_ powder sample with a fitting curve obtained by Rietveld analysis. The black “+” symbols represent the experimental pattern, and the calculated pattern is shown using red lines. The blue line represents the difference between the calculated and observed patterns. The vertical bars correspond to the positions of the Bragg reflections. Powder XRD patterns of (b) KVF_4_ and (c) K_5_V_3_F_14_. (d) Scanning electron microscopy images of K_3_VF_6_@C.

The XRD patterns of K_5_V_3_F_14_@C and KVF_4_@C are shown in Figure 2b,2c, respectively. The main peaks matched well with the previous report^25^ in both patterns. However, minor quantities of K_3_VF_6_ and VO were observed for K_5_V_3_F_14_@C, and K_5_V_3_F_14_ and minor quantities of VO were observed for KVF_4_@C.

The morphologies of carbon-coated samples, K_3_VF_6_@C, K_5_V_3_F_14_@C, and KVF_4_@C, were observed with SEM. The SEM image of K_3_VF_6_@C (Figure 2d) showed agglomerated secondary particles, 20 μm in diameter, which consist of submicrometer primary particles. The primary particles of carbon-free (uncoated) K_3_VF_6_ were also of the submicron order (Figure S3). Digital photographs comparing the color of K_3_VF_6_, before and after coating with carbon, are shown in Figure S4. The noncarbon coating was gray, whereas the carbon coating was black owing to the presence of carbon. The SEM images of K_5_V_3_F_14_@C and KVF_4_@C are presented in Figure S5a,b are similar to those of K_3_VF_6_@C.

Figure S6a,b shows the crystal structure of K_3_VF_6_ viewed along the *c*- and *a*-axes, respectively. Usually, the structure of A_*x*_M_*y*_F_*z*_ fluorides is described using the connectivity of (MF_*n*_)^*m*‑^ building units; the polyhedra share two or more vertices with the others to form chains, layers, or three-dimensional [M_*y*_F_*z*_]^*x*‑^ frameworks.^34^ Several families, including AMF_3_, AMF_4_, A_2_MF_5_, A_2_MF_6_, A_3_MF_6_, and A_2_M_2_F_7_, have been well characterized.^34,35^ However, the VF_6_ octahedra in the crystal structure of tetragonal-K_3_VF_6_ reported here do not connect with any VF_6_, which indicates that the structure is not stable without enough K^+^ ions. By contrast, as shown in Figure S7a,b, K_5_V_3_F_14_ and KVF_4_ have VF_6_ octahedra that share a corner along the *a*- and *b*-axes, respectively. Because the structural feature is correlated with the mobility of the K^+^ ion in the framework, we further compare the possible diffusion path in the structure.

The diffusion pathways of the K^+^ ions were elucidated, and their diffusion barriers were calculated by using BVEL analysis. The diffusion pathways of K_3_VF_6_ exist along all three-dimensional directions, and the diffusion barriers were calculated to be 0.77 eV along the *a-* and *b*-axes and 0.89 eV along the *c*-axis (Figure S8a). Given that the activation energy of K^+^ ions is 0.52 eV in the KTP structure,^12^ which shows excellent electrochemical properties accompanied by potassium insertion and extraction,^36^ K^+^ ions could diffuse in the K_3_VF_6_ structure.

Further, in K_5_V_3_F_14_, the K^+^ ion diffusion pathways are found in three-dimensional directions along the *a-*, *b-*, and *c*-axes, and the diffusion barriers were calculated to be 0.83, 0.83, and 0.87 eV, respectively, which are similar to those of K_3_VF_6_ (Figure S8b). For KVF_4_, the diffusion pathways of the K^+^ ions should be available only in the two-dimensional directions along the *a-* and *b*-axes; the diffusion barriers were calculated to be 1.06 and 0.10 eV, respectively (Figure S8c). Therefore, the K^+^ ions can easily diffuse in the direction along the *b*-axis, thus, we study their electrochemical potassium extraction and insertion behavior in nonaqueous K cells.

### Electrochemical K-Extraction/Insertion of K_3_VF_6_, K_5_V_3_F_14_, and KVF_4_

The galvanostatic charge and discharge (GCD) curves of synthesized K_3_VF_6_@C, K_5_V_3_F_14_@C, and KVF_4_@C using 1.0 mol dm^–3^ KPF_6_ in EC/PC (1:1 v/v) are shown in Figure 3a–c, respectively. The initial charge capacity of K_3_VF_6_@C, K_5_V_3_F_14_@C, and KVF_4_@C was ∼130, ∼190, and ∼50 mAh (g-active material including coating carbon)^−1^. It should be noted that the charge capacity must contain some irreversible capacity due to electrolyte decomposition. Nevertheless, these results indicate that a small amount of potassium (*x* < 0.3 in K_1–*x*_VF_4_) can be extracted from KVF_4_ in the range between 1.5–4.5 V (Figure 3c) probably due to the high working potential of KVF_4_. In contrast, a significant amount of potassium ions is extracted from K_3_VF_6_ (*x* ≈ 1 in K_3–*x*_VF_6_) and K_5_V_3_F_14_ (*x* ≈ 4 in K_5–*x*_V_3_F_14_). However, the initial discharge capacity of K_5_V_3_F_14_@C was ∼56 mA h (g-AM)^−1^ (Figure 3b), showing the low reversibility of the potassium insertion reaction. The low reversibility would originate from irreversible structural change during potassium extraction. By contrast, K_3_VF_6_@C exhibited an initial discharge capacity of ∼95 mAh (g-AM)^−1^ with a voltage plateau of approximately 3.7 V in the range between 2.0–4.3 V (Figure 3a). The total capacity contribution by coating and conductive agent KB was estimated to be maximum of ∼16 mAh (g-AM)^−1^ (Figure S9), and capacity contribution of K_3_VF_6_ is estimated to be ∼79 mAh (g-AM)^−1^ and ∼88 mAh (g-K_3_VF_6_)^−1^. The reversible capacity of K_3_VF_6_ almost corresponded to the capacity of a one-electron reaction (95 mAh g^–1^), suggesting the extraction/insertion of 1 mol of K^+^ ions occurs based on the V^3+/4+^ redox reaction. The K_3_VF_6_ showed a high diffusion coefficient ranging from 10^–13^ to 10^–12^ cm^2^ s^–1^ estimated by GITT measurement (Figure S10), which is competitive to Prussian blue analogues (10^–13^–10^–12^)^37^ and KVPO_4_F (10^–13^–10^–10^).^38^ By contrast, noncarbon-coated K_3_VF_6_ exhibited an initial discharge capacity of only 40 mA h g^–1^ even at a current density of C/50 in the range between 1.5–4.5 V (Figure S11), suggesting that the carbon coating is essential because of the low electronic conductivity of the material. Thus, further investigations were performed on K_3_VF_6_@C in this study.

**Figure 3:**
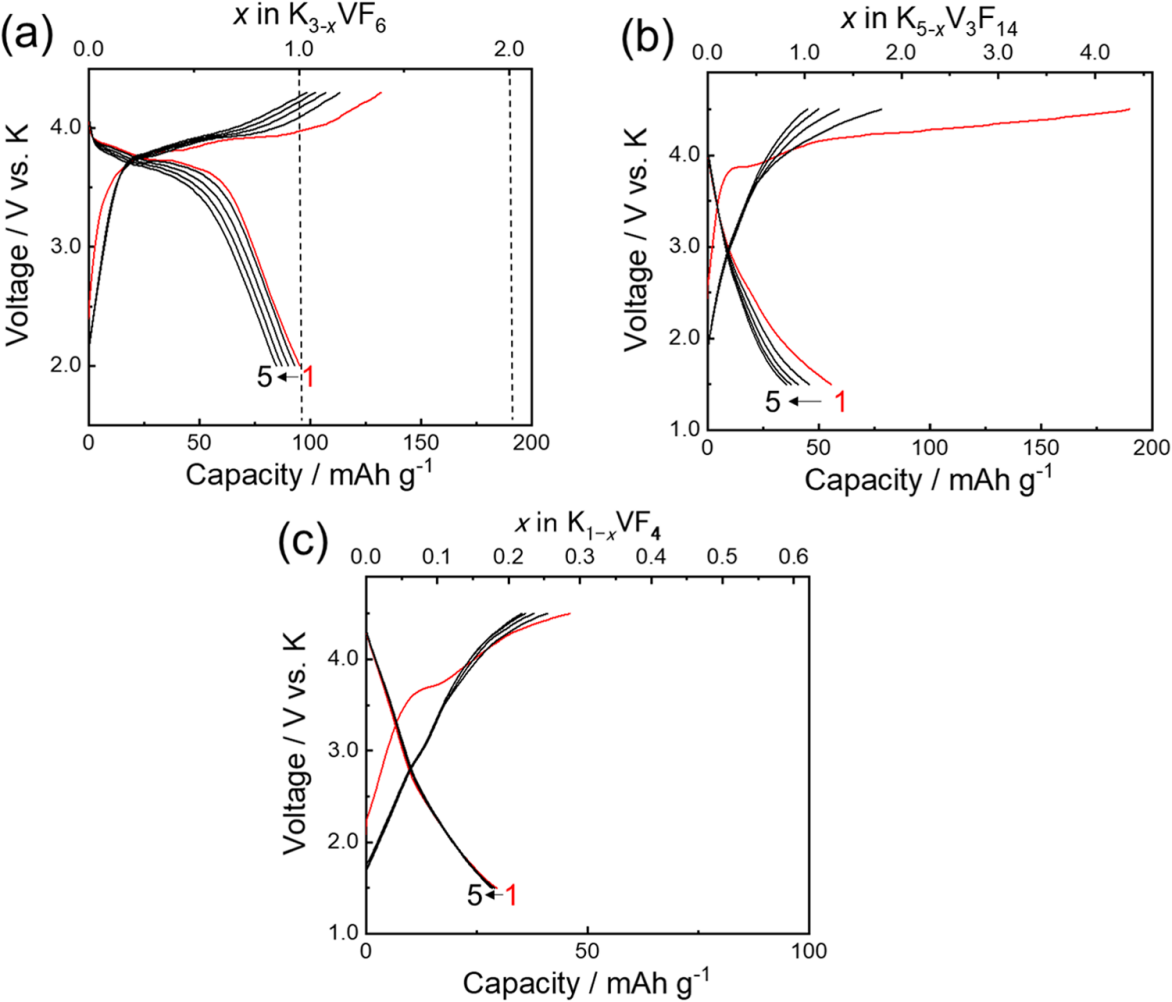
Galvanostatic charge–discharge curves of (a) K_3_VF_6_@C, (b) K_5_V_3_F_14_@C, and (c) KVF_4_@C electrodes at the C/10 current rate in the range between 2.0–4.3 V, 1.5–4.5 V, and 1.5–4.5 V, respectively, in K cells with 1.0 mol dm^–3^ KPF_6_ in EC/PC (1:1 v/v) electrolyte.

The availability of the V^4+/5+^ redox reaction was tested by raising the upper cutoff voltage to 4.6 V using a highly concentrated electrolyte (5.6 mol kg^–1^ KFSA/G3 ^39^) to suppress side reactions, which is observed in the EC/PC electrolyte at >4 V (Figure S12).^40^ This electrolyte is stable up to 4.7 V and effectively suppresses Al corrosion.^39^ Using the highly concentrated electrolyte, a second plateau appeared at approximately 4.5 V (Figure 4a), where vanadium was likely oxidized from V^4+^ to V^5+^. However, the subsequent initial discharge curve shows a negligible discharge capacity above 4 V, suggesting that the V^4+/5+^ redox reaction is irreversible. In addition, the observed capacity between 2.0–4.6 V was lower than that observed under the limited potential range of 2.0–4.3 V; this disparity could be caused by the lower ionic conductivity of the highly concentrated electrolyte.^39^ Notably, when GCD tests were performed using 5.6 mol kg^–1^ KFSA/G3 between 2.0–4.3 V, the reversible capacity decreased compared to when 1 M KPF_6_/EC/PC was used in the same voltage range (Figure S12).

**Figure 4:**
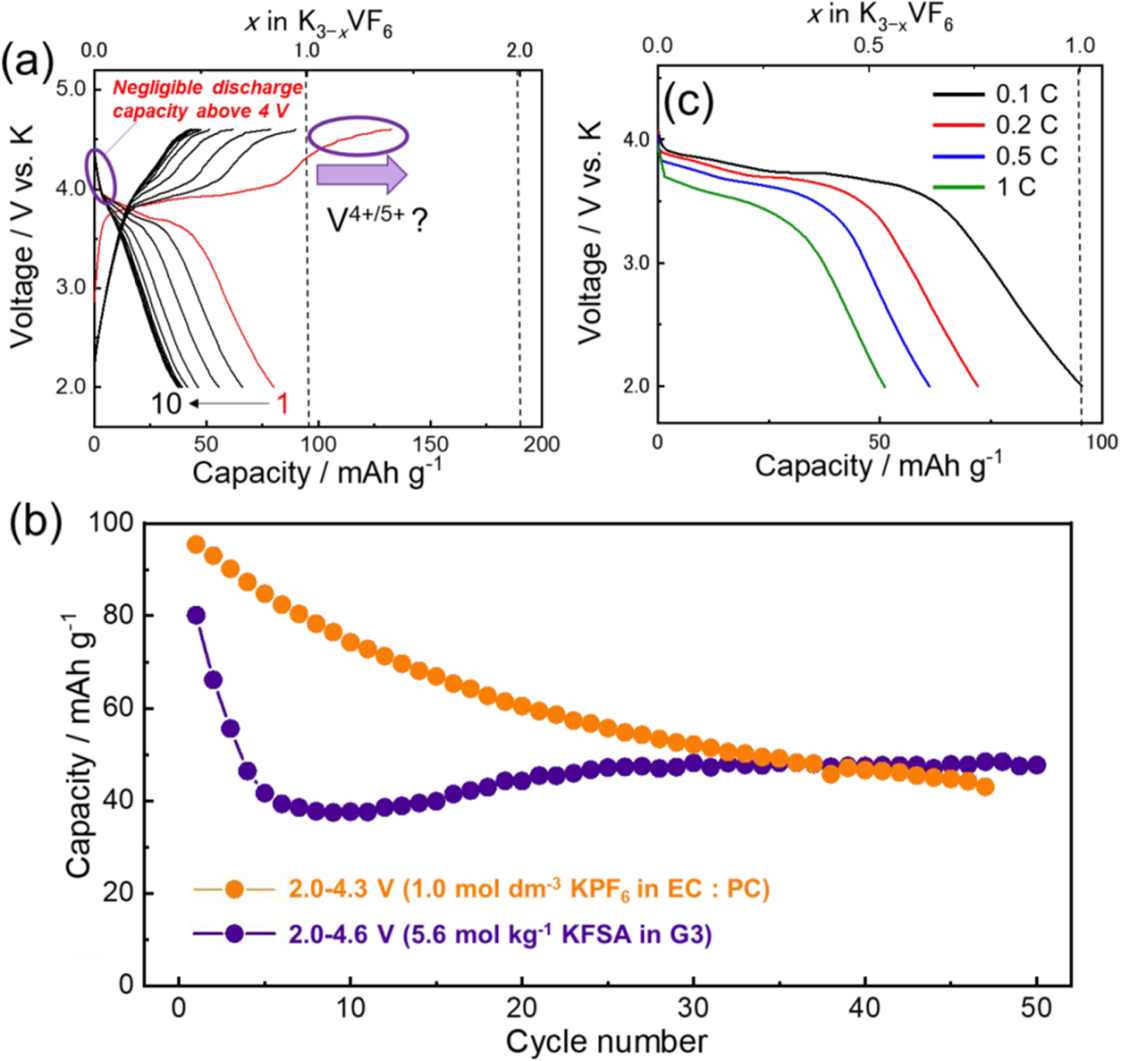
Electrochemical properties of K_3_VF_6_@C using 1.0 mol dm^–3^ KPF_6_ in EC/PC (1:1 v/v) electrolyte: (a) charge–discharge profiles in the range between 2.0 and 4.6 V, (b) capacity retention plots of K_3_VF_6_ at C/10 rate at different voltage windows, and (c) discharge profiles at different current rates during the discharging process for each cell.

For comparison, the GCD test was performed at a current density of C/10 between 1.0–3.5 V, beginning discharge from the pristine state (Figure S13), to investigate the availability of the V^3+/2+^ redox reaction. The initial discharge (potassiation) capacity was 65 mAh g^–1^; however, less capacity was obtained for the subsequent initial charge (depotassiation). The linear charge–discharge curve and low capacity suggest that only the capacitance derived from the conductive carbon is reversible. We, therefore, concluded that the reduction of V^3+^ to V^2+^ may not have proceeded or may have proceeded irreversibly with the insertion of K^+^ ions.

The capacity variation during cycling in the ranges 2.0–4.3 and 2.0–4.6 V is shown in Figure 4b. For the voltage range of 2.0–4.3 V, the discharge capacity slowly decreases during 50 cycles. By contrast, for the voltage range between 2.0 and 4.6 V, the discharge capacity decreases sharply until the fifth cycle and thereafter stabilizes at approximately 50 mAh g^–1^. The difference in the capacity fading behavior, which is probably related to structural changes during charge–discharge, will be discussed later. Moreover, highly concentrated electrolytes may have promoted the formation of stable cathode electrolyte interphases (CEI) on the electrode, which resulted in a stable capacity after a few cycles in the range between 2.0–4.6 V.^41,42^

We had hypothesized that the partial replacement of V^3+^ (ionic radius 0.640 Å) with electrochemically inert Al^3+^ (ionic radius 0.535 Å) would reduce the extent of K^+^ ion extraction/insertion, thereby suppressing the volume change during charge–discharge and improving the cycle properties. The synthesis method was the same as that used for K_3_VF_6_, with the exception that 5, 25, or 50% of the raw material, VF_3_, was replaced with AlF_3_ to synthesize K_3_V_1–*y*_Al_*y*_F_6_. XRD patterns showed that the Al-substituted sample exhibits peak shifts toward the higher angle, which would be due to the replacement of V^3+^ ions with Al^3+^ ions having a smaller ionic radius and the decrease in the interplanar spacing *d* (Figure S14). The obtained first discharge capacities were 73, 74, and 30 mA h g^–1^ for 5, 25, and 50% Al-substituted samples, respectively (Figures S14 and S15). However, the cycle performance of 5 and 25% Al-substituted samples showed a negligible improvement in cycle performance, and a gradual decrease in the reversible capacity was observed. On the other hand, 50% of Al-substitution slightly improved cycle performance over 50 cycles, though it showed less than half of the capacity compared to the other samples. Since VF_6_/AlF_6_ octahedra are connected through K^+^ ions in the crystal structure, the substitution of K^+^ ions with inactive pillar ions^43^ would improve the cycle performance, which will be discussed later.

Thereafter, the rate performance of K_3_VF_6_ was evaluated in the 2.0–4.3 V voltage range. Because K_3_VF_6_ does not exhibit excellent cycle performance (Figure 4b), a series of cells was tested with different charge–discharge rates, and their initial discharge capacities were compared. Figure 4 (c) shows the initial discharge curves at 0.1, 0.2, 0.5, and 1 C. As the current rate increases, the capacity of the material decreases, and a discharge capacity of 51 mA h g^–1^ at a rate of 1 C (190 mA g^–1^) is accomplished. The capacity obtained at the current density is comparable with that of other positive electrode materials developed for potassium batteries.^44,45^

In addition to the K system, the Li- and Na-insertion properties of K_3_VF_6_ were examined using Li//K_3_VF_6_ and Na//K_3_VF_6_ half-cells. Figure S16 shows the voltage profiles of the half-cells cycled at C/10. However, both cells displayed lower electrochemical activity and approximately half of the capacity of the K cells. Moreover, in the second cycle, both capacities deteriorated rapidly. These results indicate that K_3_VF_6_ electrodes are not suitable for Li^+^ and Na^+^ ions insertion.

We further investigated the vanadium redox during K^+^ ion extraction/insertion using an *ex-situ* X-ray absorption fine structure (XAFS). We prepared the electrodes in the charged state (4.3 V) and discharged state (2.0 V from 4.3 V), and the pristine electrode directly discharged down to 1.0 V from the pristine. The electrochemically tested electrodes were taken out by disassembling the cells for the measurements. The X-ray absorption near edge spectroscopy (XANES) spectra of the V K-edge are shown in Figure 5 (a) and a magnified view of the pre-edge region, which is sensitive to both local symmetry and oxidation states,^46,47^ is shown in Figure 5 (b). The pristine K_3_VF_6_ showed very low pre-edge peak intensity, indicating V^3+^ in octahedral symmetry.^46,47^ When charging up to 4.3 V, the absorption edge shifted to a higher energy and the pre-edge peak intensity became stronger than that of the pristine sample, clearly indicating a change in oxidation from V^3+^ to V^4+^.^16,47^ Then, upon discharging down to 2.0 V, the spectrum profile returned to a profile similar to that of its pristine state, suggesting that V^4+^ was reduced to V^3+^ during the discharge process. These results indicate that the redox reaction of V^3+/4+^ is reversible during charge–discharge in the range between 2.0–4.3 V.

**Figure 5:**
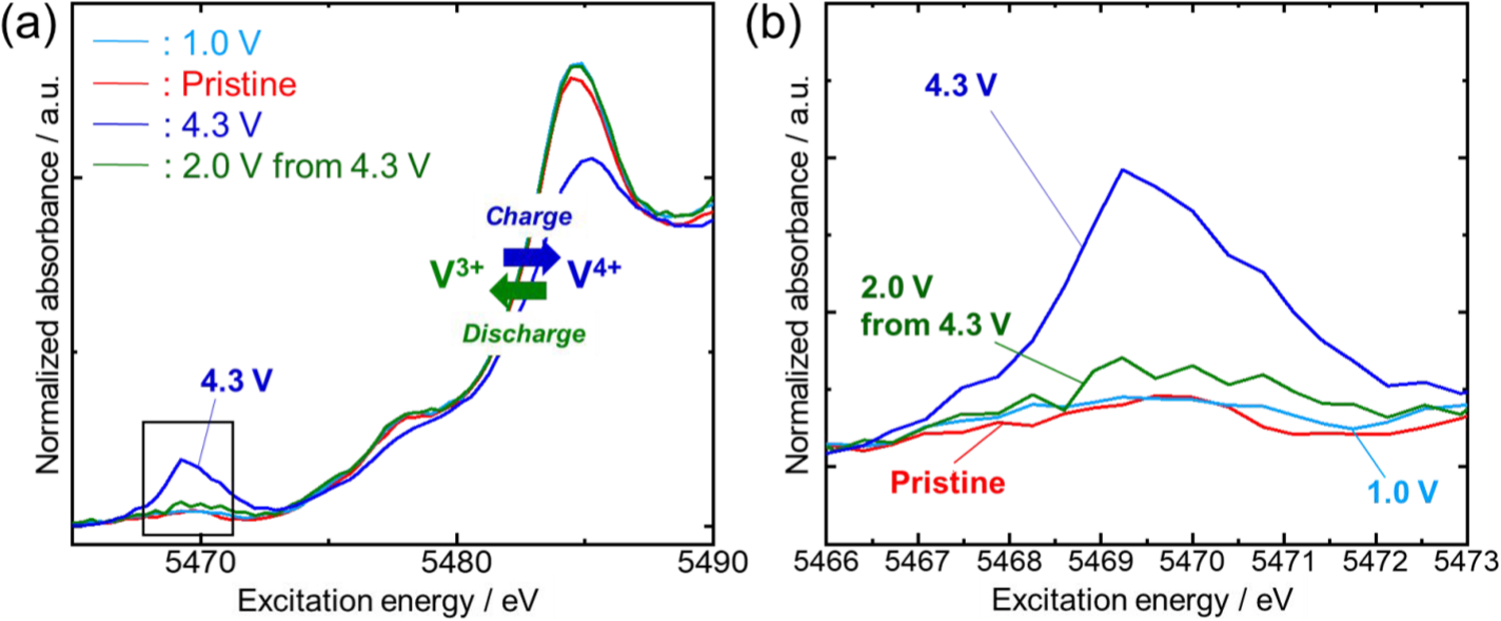
(a) *Ex-situ* X-ray absorption near edge spectroscopy spectra of the vanadium K-edge for the pristine and electrochemically tested K_3_VF_6_ electrodes in K cells with 1.0 mol dm^–3^ KPF_6_ in EC/PC (1:1 v/v) electrolyte. (b) Magnified view of pre-edge spectra.

The spectrum obtained after discharging to 1.0 V showed an absorption edge almost identical with that of the pristine state, suggesting no reduction from V^3+^ to V^2+^. Actually, we visually confirmed the yellow hue of the separator taken from the 1.0 V discharged cell (Figure S17), which is indicative that the initial discharge capacity (Figure S13) obtained during discharging to 1.0 V was due to side reactions such as active material or electrolyte decomposition.^48^ By comparing the diffraction patterns of discharged to 1.0 V and the pristine samples (Figure S18), no obvious difference was found, indicating no change in the long-range order and lattice constant, that is, the K^+^ ion insertion reaction did not proceed during the discharge process down to 1.0 V, and obtained irreversible capacity can be attributed to side reactions. This result is consistent with the fact that the valence of vanadium was not reduced from the trivalent state in the XAFS measurement described above (Figure 5).

SEM-EDS measurements were performed to examine the change in potassium content in the tested K_3_VF_6_ electrodes during the charge/discharge processes (Figure 6). The composition was determined by quantifying the relative intensity of each state from the intensity of the K–Kα peak in the EDS spectra. The maximum value of the relative intensity was set to 1.0 (corresponding to K_3_VF_6_). The relative intensity decreased from 1.0 to 0.65 and 0.42 upon charging to 4.3 and 4.6 V, which correspond to K_2.0_VF_6_ and K_1.3_VF_6_, respectively. Although we failed to measure the XAFS of the 4.6 V charged sample, the EDS results suggest partial vanadium oxidation to pentavalent. An increase in K was observed upon discharging to 2.0 V from 4.3 V, which corresponds to K_3.0_VF_6_ and, respectively. These results agree with the above discussion and indicate that one K^+^ ion extraction/insertion per formula based on the V^3+/4+^ redox was reversible, as confirmed in the XAFS measurements. On the other hand, K content was smaller (K_2.1_VF_6_) when the sample was discharged to 2.0 V from 4.6 V, indicating K^+^ ions were not fully reinserted after the electrode was charged >4.3 V, which will be discussed in the next section.

**Figure 6:**
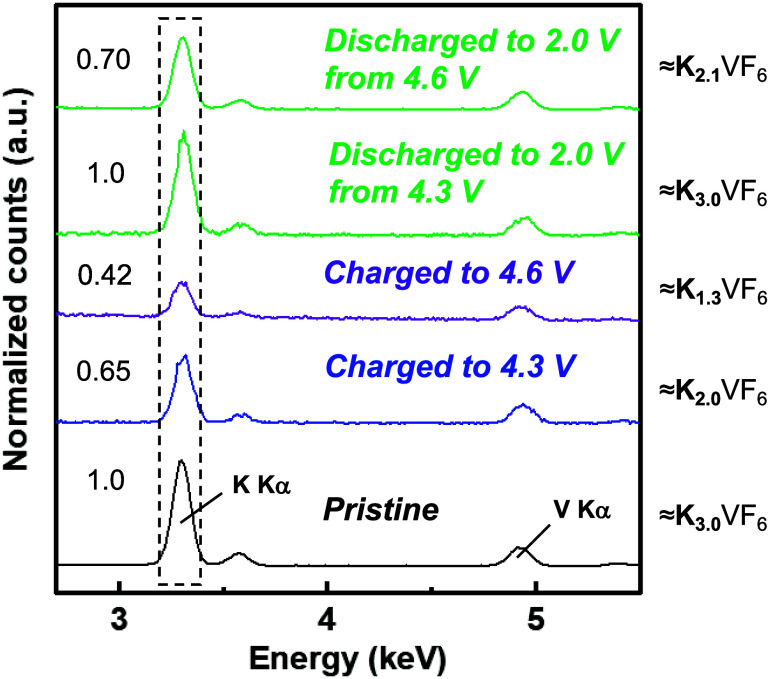
Energy-dispersive X-ray spectroscopy spectra of pristine and tested K_3_VF_6_@C electrodes after charging to 4.3 V, charging to 4.6 V, and discharging to 2.0 V states using 1.0 mol dm^–3^ KPF_6_ in the EC/PC (1:1 v/v) electrolyte. Peak intensities are normalized with V Kα.

### Structural Evolution of K_3_VF_6_

Because of the reversible electrode properties, *ex-situ* SXRD measurements were performed in both voltage ranges to understand the structural evolution of K_3_VF_6_ during K-extraction/insertion, as shown in Figure 7a. The lattice constant obtained by Le Bail fitting (Figures S19 and 7b) decreases during charge. Moreover, several peaks, such as 150, 510, and shoulder peaks, disappear after being charged up to 4.3 V (Figure S21a,b), suggesting a structural transition from tetragonal to cubic with decreasing lattice distortion. Indeed, the SXRD pattern of the 4.3 V-charge sample was successfully fitted by the Rietveld method (Figure S20 and Table S2) with a cubic system of space group *Fm3̅m* (225), which is isomorphous to K_2_MnF_6_.^49^ Overall, the volume change from K_3_VF_6_ to K_2_VF_6_ was −6.4%. By further charging to 4.6 V, which results in an additional 0.7 mol K extraction per formula to K_1.3_VF_6_ (Figure 6), the peak positions of the main peaks were almost constant (Figures 7a and S21a), whereas several new peaks appeared (Figures 7a and S21c), suggesting additional phase evolution.

**Figure 7:**
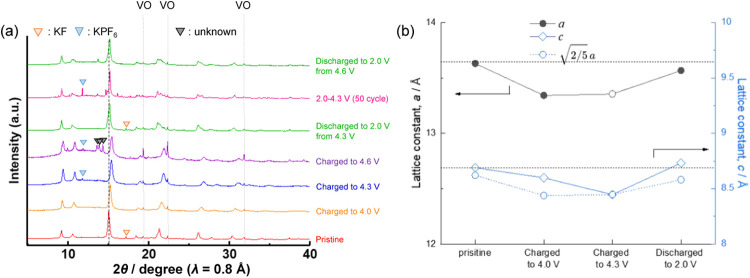
(a) *Ex-situ* synchrotron X-ray diffraction patterns of pristine and tested K_3_VF_6_ electrodes; first charged up to 4.0, 4.3, and 4.6 V, first discharged to 2.0 V from 4.3 V, 50th discharge during cycling in 2.0–4.3 V, first discharged to 2.0 V from 4.6 V, 50th discharge to 2.0 V (2.0–4.6 V). (b) Evolution of lattice constant during the first charge–discharge process.

Upon discharge from 4.3 to 2.0 V, the sample almost returned to its original tetragonal structure (Figures 7a and S21a). However, we observed minor peak shifts compared to those of the pristine electrode. Specifically, *hk*0 diffraction peaks shift to higher angles, and 00*l* diffraction peaks shift to slightly lower angles (Figure S21d,e). In other words, lattice constant *a* decreased, whereas *c* increased after one cycle compared to the pristine electrode (Figure 7b). This means an increase in lattice distortion, as shown in the increased difference between *c* and $\sqrt{2/5}$ *a*, which is equal to *c* when there is no lattice distortion (Figure 7b). The irreversible peak shifts were also observed the electrode cycled in 2.0 V–4.6 V. In addition, several new peaks, e.g., at 13.6 and 13.9°, that cannot be assigned to the original structure appeared, and a few peaks, such as 150/510 of the original tetragonal phase, disappeared for the electrode cycled in 2.0 V–4.6 V (Figure S21c), indicating an irreversible structural change. Since this irreversibility was insignificant for the electrode cycled in 2.0 V–4.3 V, this is mainly due to the structural evolution above 4.3 V (*x* > 1 in K_3–*x*_VF_6_). Although further study is essential to fully understand the structural evolution at high voltage, the irreversible structural evolution after K^+^ ion extraction can be attributed to the structure characteristic of K_3_VF_6_ where VF_6_ octahedra are connected through K^+^ ions. Thus, the substitution of K^+^ ions with inactive cations, *i.e*., “pillar” ions,^43^ will be an effective strategy to stabilize the framework and avoid capacity degradation.

## Conclusions

In this study, we investigated the electrochemical behavior of potassium vanadium fluorides as promising positive electrode materials for KIBs. We found that K_5_V_3_F_14_ and KVF_4_ exhibit low electrochemical activity with ∼50 mAh g^–1^ and that K_3_VF_6_ exhibited a voltage plateau at approximately 3.7 V during discharge in the range between 2.0–4.3 V and a discharge capacity of approximately 95 mA h g^–1^. XAFS measurements revealed that the reversible V^3+/4+^ redox reaction was responsible for the capacity. By contrast, the measurements suggested that V^4+/5+^ is irreversible and V^3+/2+^ is inactive. A series of *ex-situ* SXRD measurements revealed a partially irreversible structural evolution during potassium insertion/extraction, especially when more than 1 mol of potassium ions were extracted from K_3_VF_6_. The irreversible structural change would be the main reason for the capacity degradation and limited capacity. These findings indicate that structural stability after potassium extraction is essential in designing high-capacity and long-life fluoride materials for KIBs.

## Supplementary Material



## Notes

XRD patterns of VF_3_ material; SEM images of K_5_V_3_F_14_/C-15, KVF_4_/C-20, and K_3_VF_6_ without carbon coated; digital photos of powders for K_3_VF_6_ without carbon coated and carbon coated; schematic illustrations of the crystal structures for K_3_VF_6_, K_5_V_3_F_14_, and KVF_4_; diffusion barrier of K^+^ ion in K_5_V_3_F_14_, K_3_VF_6_, and KVF_4_ structure; GCD curves of K_3_VF_6_ without carbon-coated electrodes; GCD curves of K_3_VF_6_ in different electrolytes; GCD curves of K_3_VF_6_ electrodes in the range of 1.0–3.5 V; comparison of Non substitution and Al substitution for XRD patterns. GCD curves of K_3_V_0.75_Al_0.25_F_6_ electrodes; GCD curves of K_3_VF_6_ electrodes in Li and Na half-cells; the digital photo of the separator for being discharged to 1.0 V; comparison of laboratory raw XRD patterns of pristine K_3_VF_6_ electrode and first discharged to 1.0 V electrode; synchrotron XRD pattern of first charged to 4.3 V for K_3_VF_6_; and atomic positions of pristine K_3_VF_6_ and first charged to 4.3 V for K_3_VF_6_ (PDF)

## Abbreviations

BVS: bond valence sum
EDS: energy-dispersive X-ray spectroscopy
GCD: galvanostatic charge and discharge
ICP: inductively coupled plasma spectroscopy
KB: ketjen black
LIB: lithium-ion batteries
SEM: scanning electron microscopy
SXRD: synchrotron X-ray diffraction
XAFS: X-ray absorption fine structure
XANES: X-ray absorption near edge spectroscopy
XAS: X-ray absorption spectroscopy
XRD: X-ray diffraction
