# Supplementary material for: Potassium Vanadium Fluorides as Positive Electrode Materials for K‑ion Batteries
Source: ACS Appl Mater Interfaces. 2025 May 7;17(20):29610–8. doi: 10.1021/acsami.5c02298 (PMC12100645; doi:10.1021/acsami.5c02298)
Supplement: Supplementary file 1 [file am5c02298_si_001.pdf]

Supporting Information for

Potassium vanadium fluorides as positive electrode materials for K-ion batteries

Kazushi Magara, Tomooki Hosaka, Ryoichi Tatara, Shinichi Komaba[a]

AUTHOR ADDRESS

Department of Applied Chemistry, Tokyo University of Science, 1-3 Kagurazaka,

Shinjuku-ku, Tokyo 162-8601, Japan. E-mail: komaba@rs.tus.ac.jp

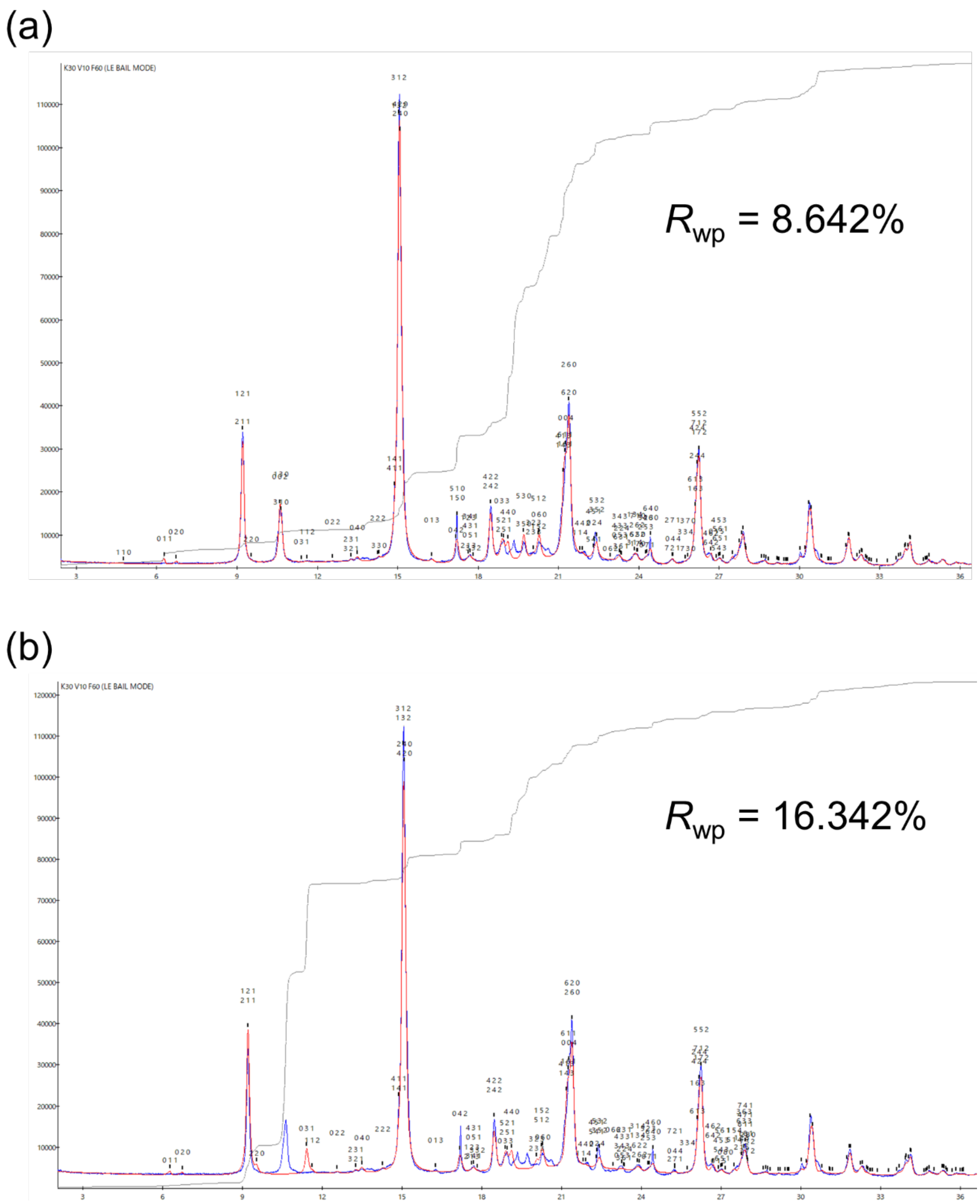

Figure S1. Le Bail fittings of SXRD pattern of K_3VF_6 with (a) *I*4/*m* and (b) *I*4$_1$/*a* space groups.

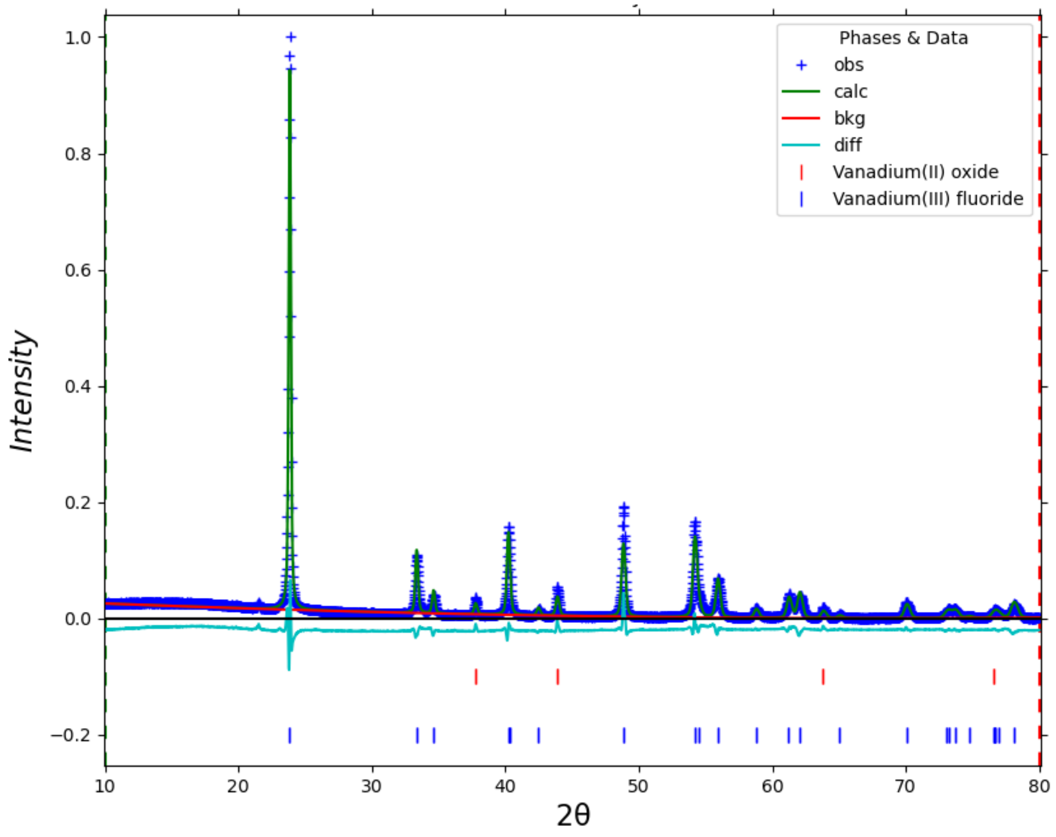

Figure S2. XRD patterns of the VF_3 and fitting curves by Rietveld method using two phases of VF_3 and VO.

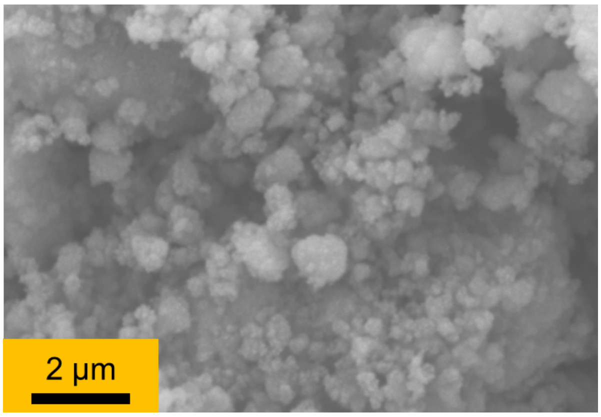

Figure S3. SEM images of K_3VF_6 without carbon coating.

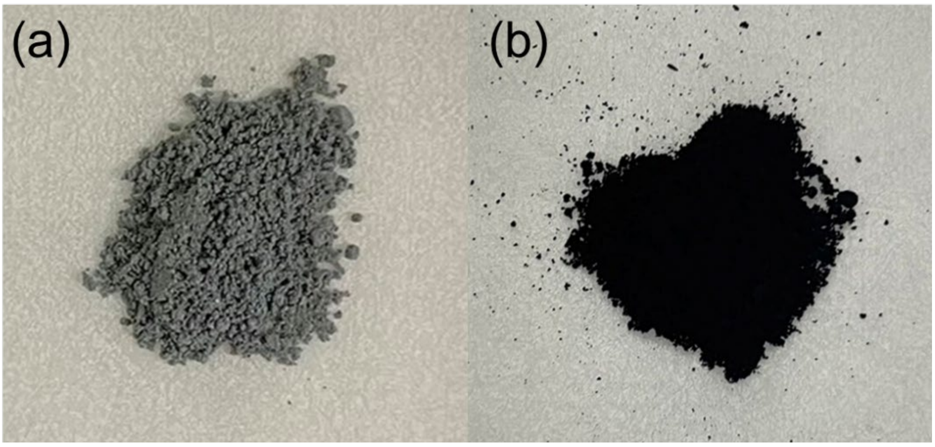

Figure S4. Digital photos of powders for (a) K_3VF_6 without carbon coating and (b) with carbon coating.

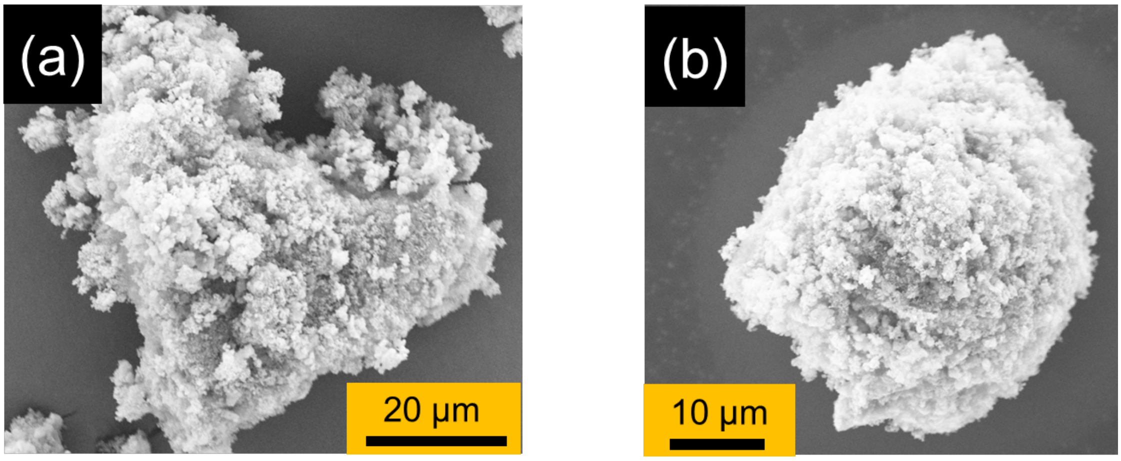

Figure S5. SEM images of (a) $K_5V_3F_{14}$/C-15 and (b) KVF_4/C-20.

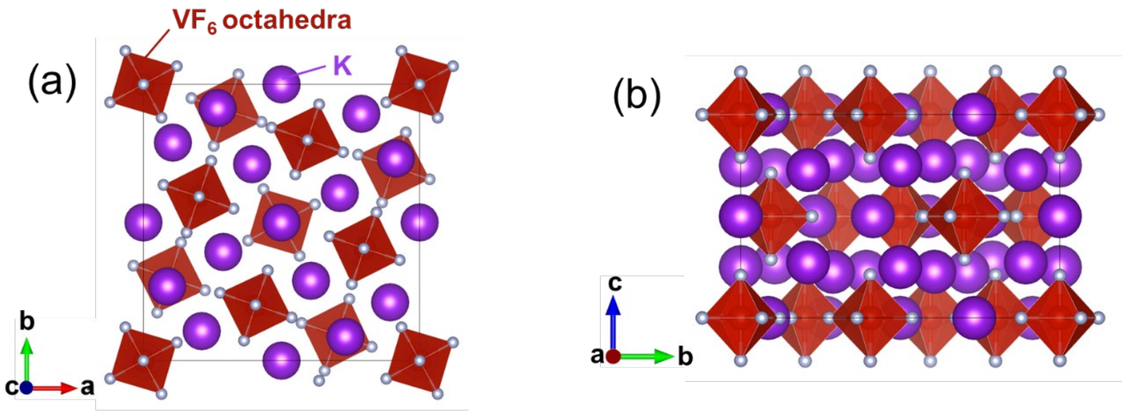

Figure S6. Schematic illustrations of the crystal structures for K_3VF_6 projected along (a) c-axis and (b) a-axis.

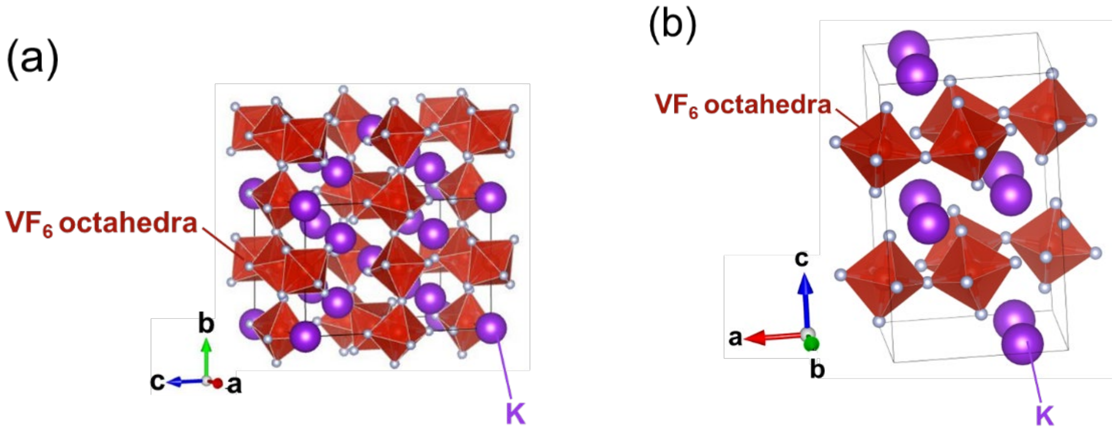

Figure S7. Schematic illustrations of the crystal structures for (a) $K_5V_3F_{14}$ and (b) KVF_4.

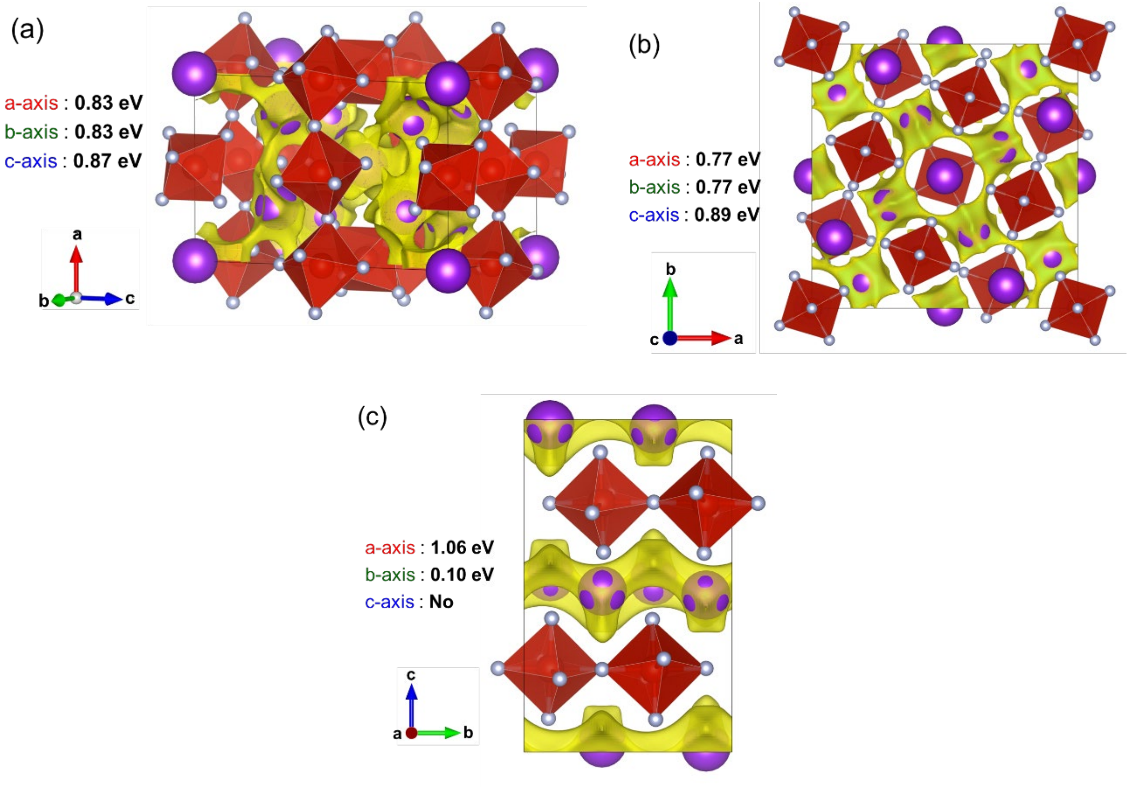

Figure S8. Diffusion barrier of K^+ ion in (a) $K_5V_3F_{14}$, (b) K_3VF_6 and (c) KVF_4 structure. Diffusion barrier is obtained by bond valency energy landscape (BVEL) method.

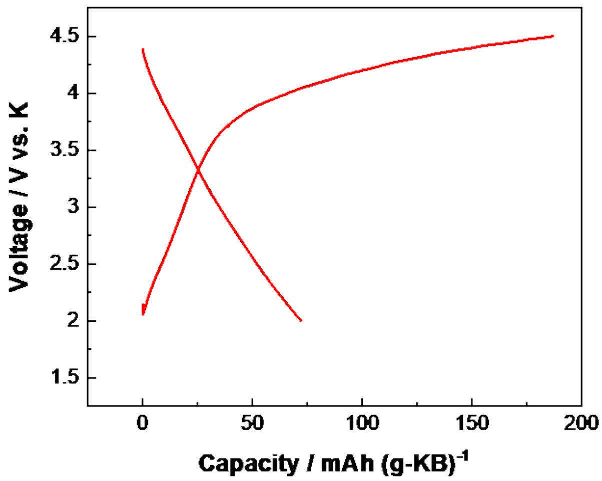

Figure S9. Charge-discharge curve of KB:PVdF (7:3 wt. ratio) electrode in 1.0 mol dm^{-3} KPF_6 / EC:PC (1:1 v/v) electrolyte in the voltage range of 2.0–4.5 V.

The obtained capacity was 72 mAh $(g\text{-}KB)^{-1}$, which was used to estimate the KB contribution to the capacity (mAh g_{AM}^{-1}). We assumed that the coating KB in the active material and conductive KB in

the electrode show the same capacity. The contribution of coating KB ($C_{\text{KB\_coating}}$) was calculated by Equation S1, where $r_{\text{KB\_AM}}$ is the weight ratio of KB in the active material (0.1 for K_3VF_6@C).

$$C_{\text{KB\_coating}} = 72\ (\text{mAh g}_{\text{KB}}^{-1}) \times r_{KB_{AM}} \left(\frac{g_{\text{KB}}}{g_{\text{AM}}}\right) \qquad (\text{S1})$$

The capacity of the conductive KB ($C_{\text{KB\_conductive}}$) was estimated by Equation S2, where $r_{\text{KB\_electrode}}$ is the weight ratio of the KB as conductive agent against the active material (1/8).

$$C_{\text{KB\_conductive}} = 72\ (\text{mAh g}_{\text{KB}}^{-1}) \times r_{\text{KB}_{\text{electrode}}} \left(\frac{g_{\text{KB}}}{g_{\text{AM}}}\right) \qquad (\text{S2})$$

Then, the $\boldsymbol{C}_{\textbf{KB\_coating}}$ and $C_{\text{KB\_conductive}}$ were used to calculate the capacity of K_3VF_6 per active material (K_3VF_6@C) or per K_3VF_6 mass using Equation S3 or S4, respectively, where r_{KVF} is the ratio of K_3VF_6 in the active material (9/10).

$$C_{KVF_{AM}} = C_{obs} - C_{\text{KB}_{\text{coating}}} - C_{\text{KB}_{\text{conductive}}} \qquad (\text{S3})$$

$$C_{\text{KVF}_{\text{KVF}}} = \frac{C_{\text{KVF}_{\text{AM}}}}{r_{\text{KVF}}} \qquad (\text{S4})$$

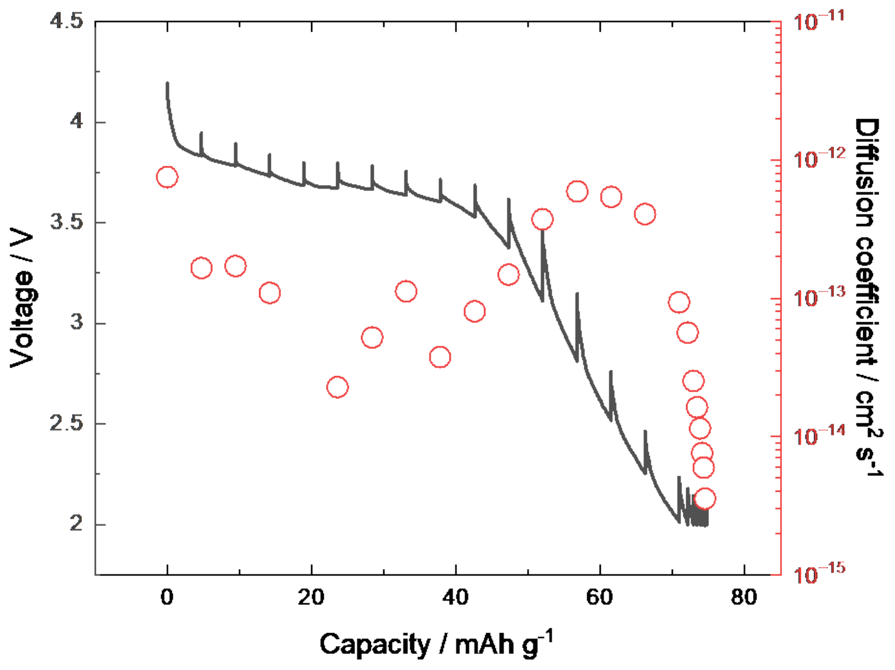

Figure S10. GITT curves and calculated diffusion coefficient at the first discharge process. Before the GITT measurement, the cell was charged with CC mode at C/20. Then, the electrodes were discharged at C/20 current pulse for 15 min and rested for 1 h. The relaxation time was selected to minimize the self-discharge effect. The K^+ ion diffusion coefficient was calculated using an equation

reported previously. [1] Note that diffusion coefficients in the plateau region can be apparent diffusion coefficients due to a two-phase reaction.[2]

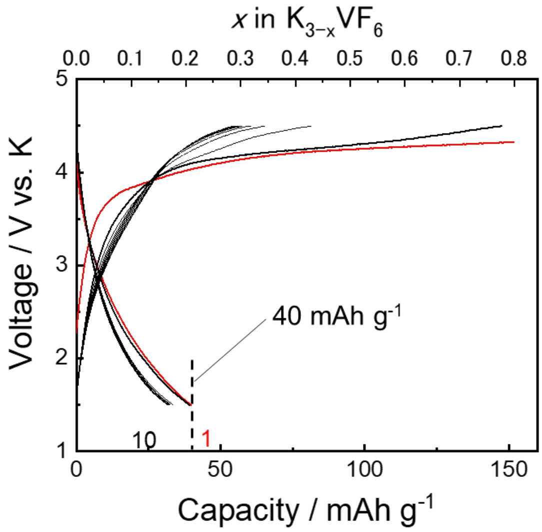

Figure S11. Galvanostatic charge-discharge curves of K_3VF_6 without carbon-coated electrodes at C/50 current rate in the range of 1.5-4.5 V in K cells.

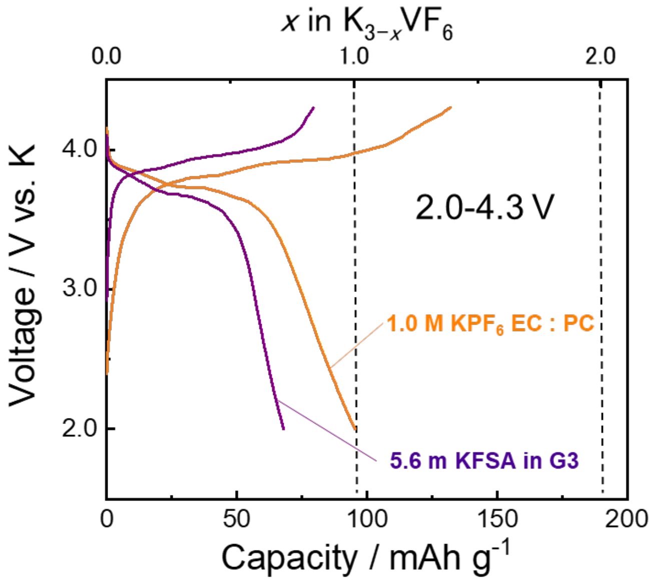

Figure S12. Galvanostatic charge-discharge curves of K_3VF_6 in 1.0 M KPF_6/EC : PC or 5.6 m KFSA/G3.

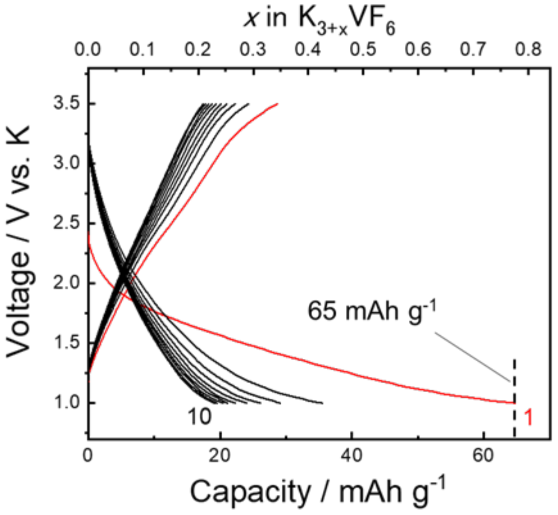

Figure S13. Galvanostatic charge-discharge curves of K_3VF_6 electrodes at C/10 current rate in the range of 1.0-3.5 V in K cells.

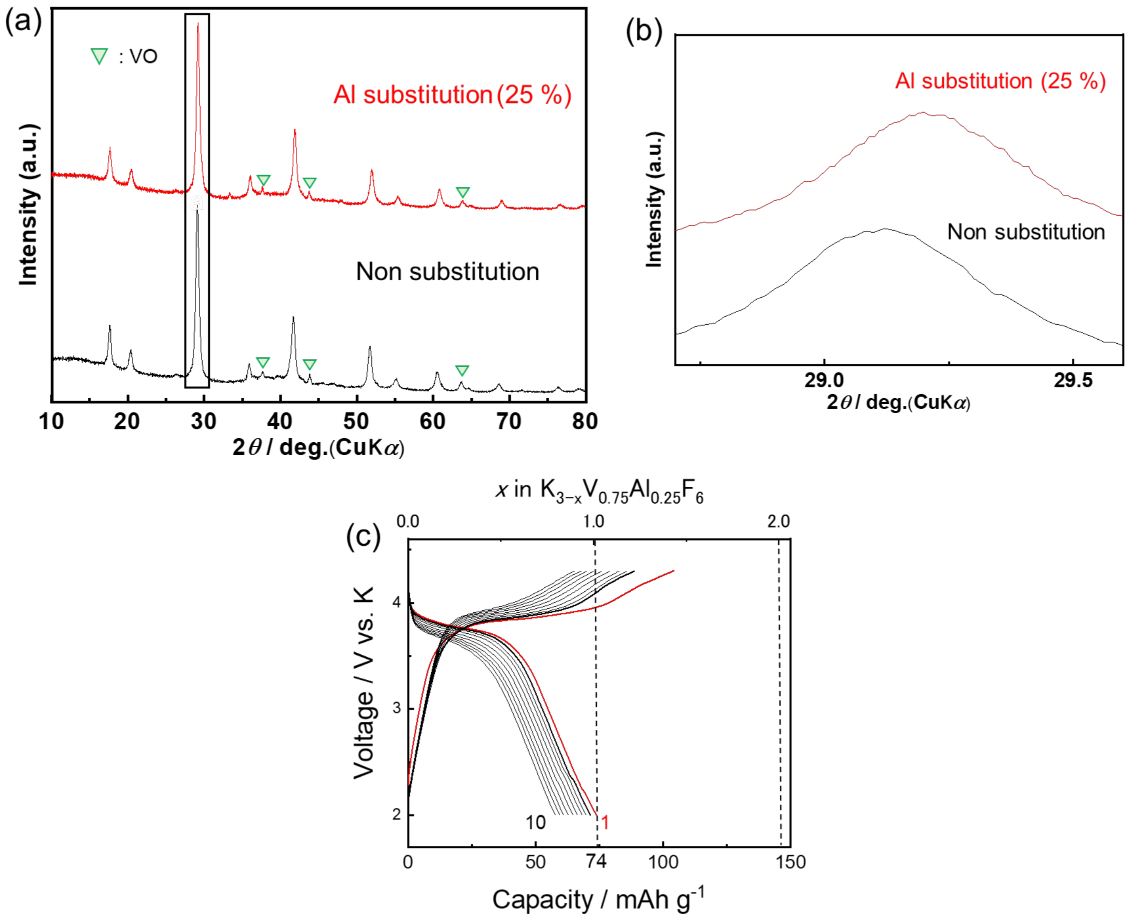

Figure S14. (a) (b) Comparison of K_3VF_6 (Non substitution) and $K_3V_{0.75}Al_{0.25}F_6$ (Al substitution) for XRD patterns. (c) Galvanostatic charge-discharge curves of $K_3V_{0.75}Al_{0.25}F_6$ electrodes at C/10 current rate in the range of 2.0-4.3 V in K cells.

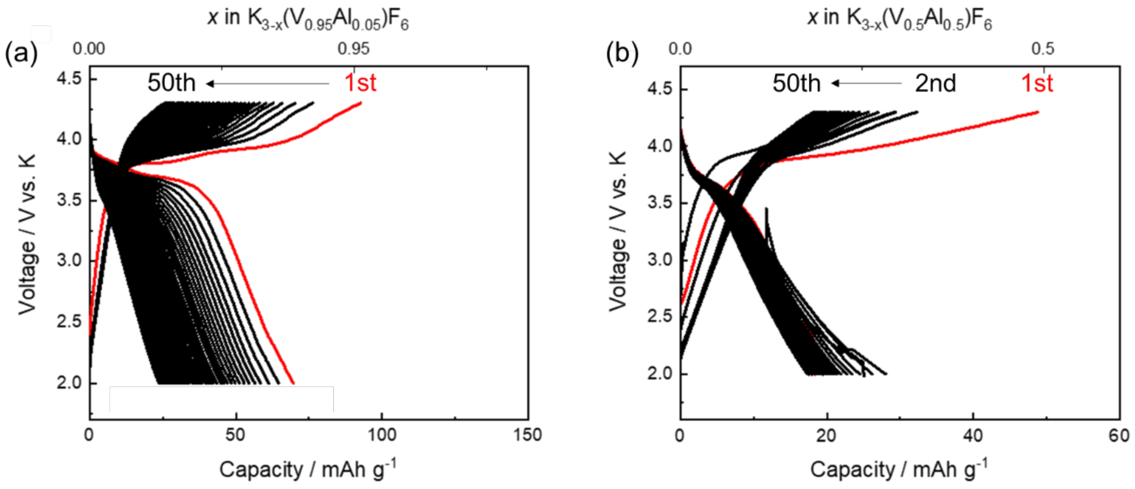

Figure S15. Galvanostatic charge-discharge curves of (a) $K_3V_{0.95}Al_{0.05}F_6$ and (b) $K_3V_{0.5}Al_{0.5}F_6$ electrodes at C/10 current rate in the range of 2.0-4.3 V in K cells.

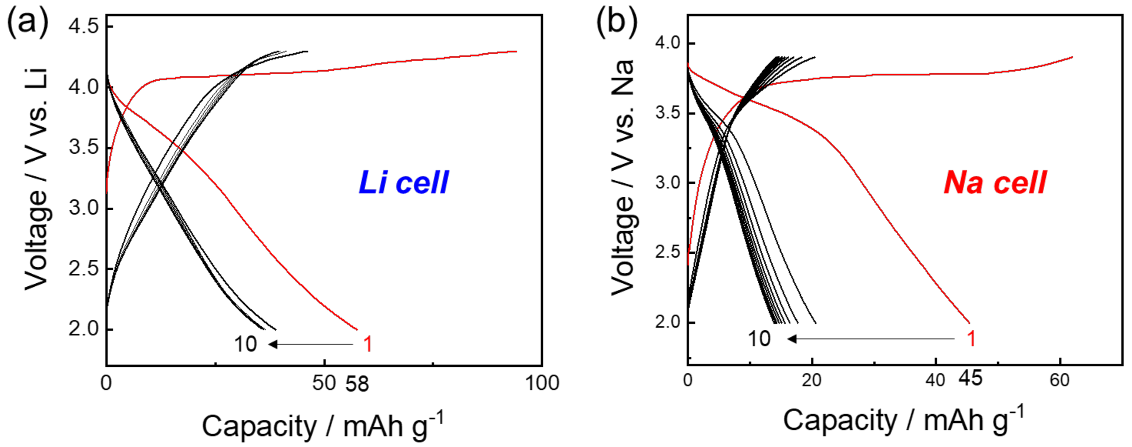

Figure S16. Charge-discharge curves of K_3VF_6 electrodes in (a) Li and (b) Na half-cells tested for first 10 cycles in the range of 2.0-4.3 V and 2.0-3.9 V, respectively.

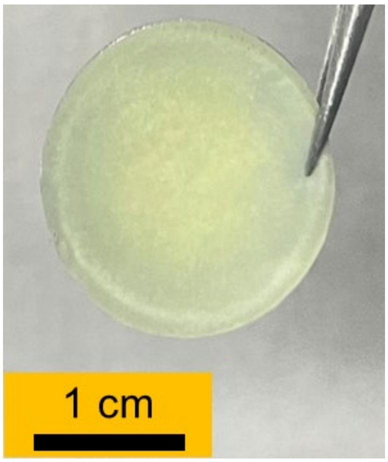

Figure S17. The digital photo of the separator for being discharged to 1.0 V

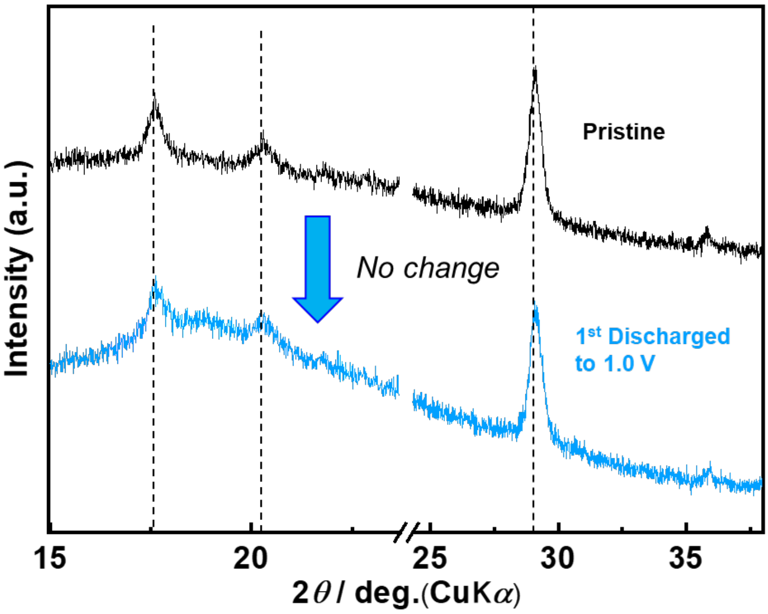

Figure S18. Comparison of XRD patterns of pristine K_3VF_6 electrode and the electrode discharged to 1.0 V electrode.

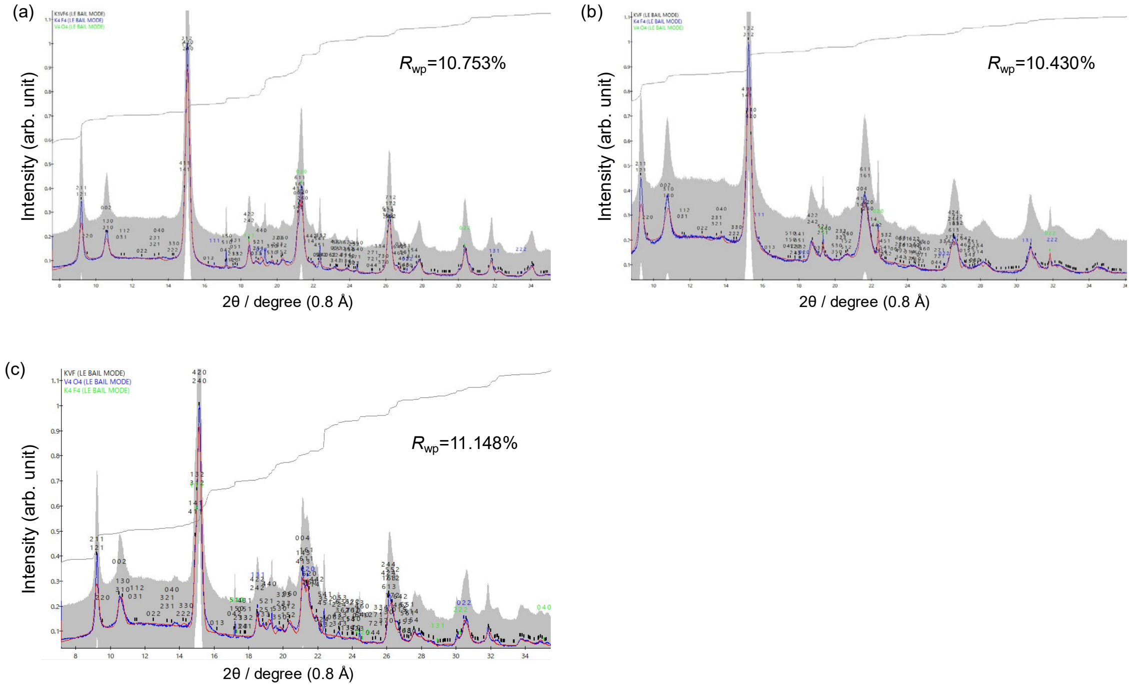

Figure S19. Le Bail fittings of SXRD patterns during the first charge-discharge process: (a) pristine, (b) charged to 4.0 V, and (c) discharged to 2.0 V from 4.3 V.

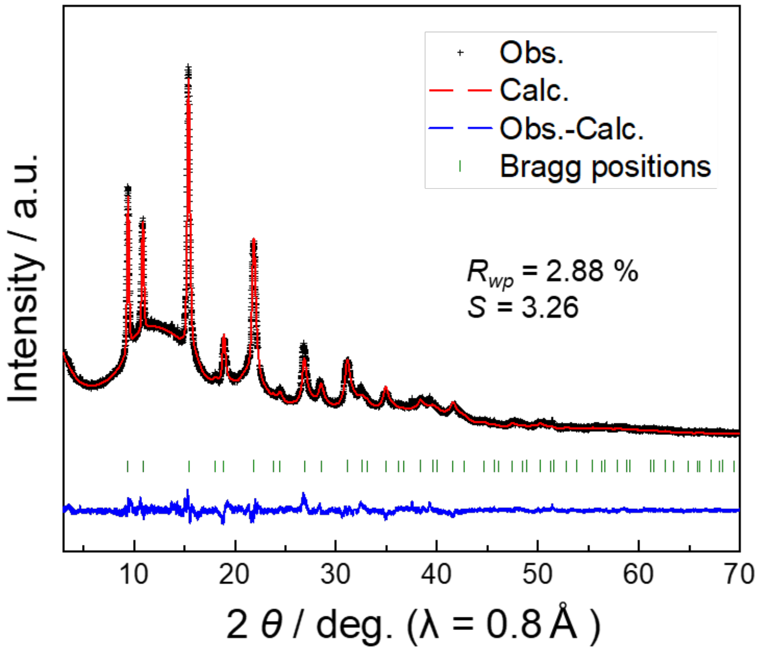

Figure S20. SXRD pattern of the K_3VF_6 charged to 4.3 V and fitting pattern by Rietveld method.

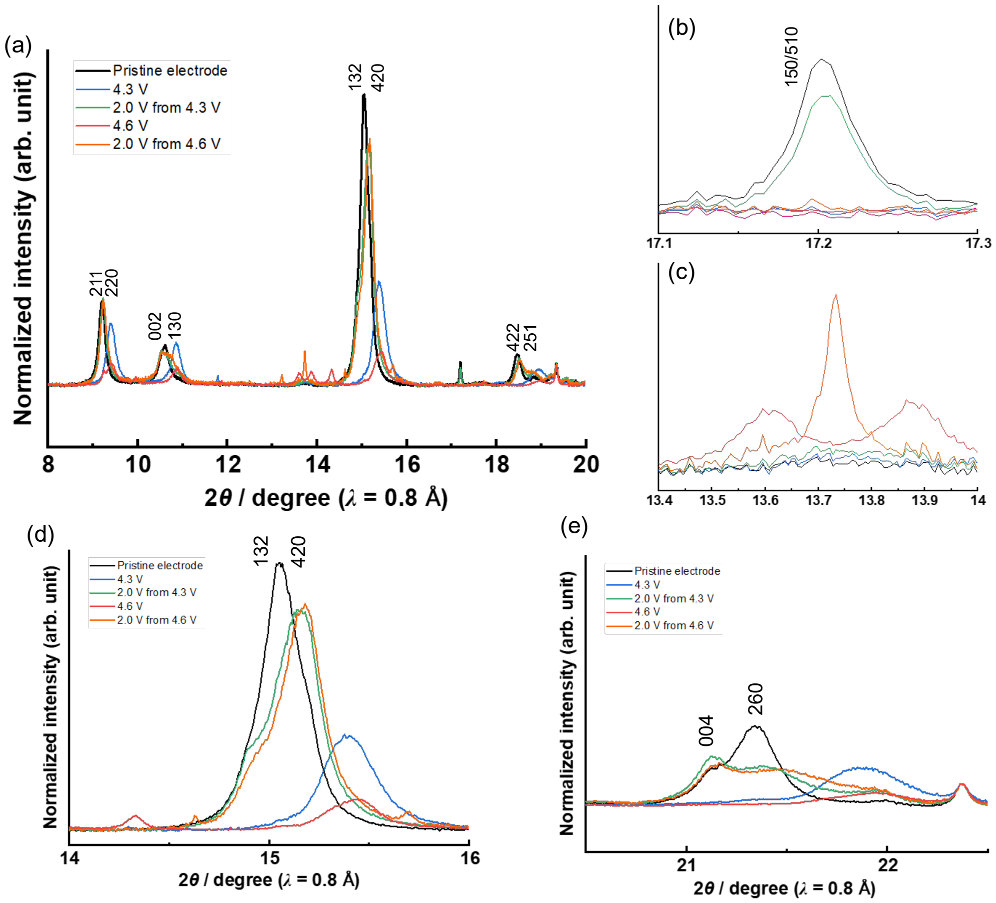

Figure S21. Comparison of ex-situ XRD patterns of K_xVF_6 electrodes: (a) range of 2θ = 8°–20°, (b) enlarged figure around 2θ = 17.2°, (c) 2θ = 13.7°, (d) 2θ = 15.0°, and (d) 2θ = 21.5°.

Table S1. Atomic positions of pristine K_3VF_6.

S.G. *I*4/*m* (87), a = b = 13.6262, c = 8.66654, α = β = γ = 90°						
Atom	site	x	y	z	Occ.	B
K1	16i	0.0954905	0.787896	0.272783	1.0	0.7
K2	8h	0.108959	0.256281	0.0	1.0	0.7
K3	4d	0.0	0.5	0.25	1.0	0.7
K4	2b	0.0	0.0	0.5	1.0	0.7
V1	8h	0.10043	0.29325	0.5	1.0	0.5
V2	2a	0.0	0.0	0.0	1.0	0.5
F1	16i	0.102064	0.298962	0.281188	1.0	0.7
F2	8h	0.0497608	0.671529	0.5	1.0	0.7
F3	8h	0.0664502	0.195964	0.5	1.0	0.7
F4	8h	0.0739439	0.883309	0.0	1.0	0.7
F5	8h	0.0505721	0.631831	0.0	1.0	0.7
F6	8h	0.219583	0.343873	0.5	1.0	0.7
F7	4e	0.0	0.0	0.226534	1.0	0.7

Table S2. Atomic positions of 1st charged to 4.3 V for K_3VF_6.

S.G. *Fm-3m* (225), a = b = c = 8.44592, α = β = γ = 90°						
Atom	site	x	y	z	Occ.	B
K1	8c	0.25	0.25	0.25	0.769808	1.0
K2	4b	0.5	0.5	0.5	0.677169	1.0
V1	4a	0.0	0.0	0.0	1.0	0.5
F1	24e	0.21301	0.0	0.0	0.87747	0.7

References:

(1) Nickol, A.; Schied, T.; Heubner, C.; Schneider, M.; Michaelis, A.; Bobeth, M.; Cuniberti, G. GITT Analysis of Lithium Insertion Cathodes for Determining the Lithium Diffusion Coefficient at Low Temperature: Challenges and Pitfalls. *J. Electrochem. Soc.* **2020**, *167* (9), 090546. https://doi.org/10.1149/1945-7111/ab9404.

(2) Zhu, Y.; Wang, C. Galvanostatic Intermittent Titration Technique for Phase-Transformation Electrodes. *J. Phys. Chem. C* **2010**, *114* (6), 2830–2841. https://doi.org/10.1021/jp9113333.
